# Thresholding functional connectomes by means of mixture modeling

**DOI:** 10.1016/j.neuroimage.2018.01.003

**Published:** 2018-05-01

**Authors:** Natalia Z. Bielczyk, Fabian Walocha, Patrick W. Ebel, Koen V. Haak, Alberto Llera, Jan K. Buitelaar, Jeffrey C. Glennon, Christian F. Beckmann

**Affiliations:** a Donders Institute for Brain, Cognition and Behaviour, Centre for Cognitive Neuroimaging, Nijmegen, The Netherlands; b Department of Cognitive Neuroscience, Radboud University Nijmegen Medical Centre, Geert Groteplein Zuid 10, 6525GA Nijmegen, The Netherlands; c University of Osnabrück, Neuer Graben 29/Schloss, 49074 Osnabrück, Germany; d Radboud University Nijmegen, Comeniuslaan 4, 6525 HP Nijmegen, The Netherlands; e Oxford Centre for Functional MRI of the Brain, John Radcliffe Hospital, Oxford OX3 9DU, United Kingdom

**Keywords:** Functional connectivity, Mixture modeling, False discovery rate

## Abstract

Functional connectivity has been shown to be a very promising tool for studying the large-scale functional architecture of the human brain. In network research in fMRI, functional connectivity is considered as a set of pair-wise interactions between the nodes of the network. These interactions are typically operationalized through the full or partial correlation between all pairs of regional time series. Estimating the structure of the latent underlying functional connectome from the set of pair-wise partial correlations remains an open research problem though. Typically, this thresholding problem is approached by proportional thresholding, or by means of parametric or non-parametric permutation testing across a cohort of subjects at each possible connection. As an alternative, we propose a data-driven thresholding approach for network matrices on the basis of mixture modeling. This approach allows for creating subject-specific sparse connectomes by modeling the full set of partial correlations as a mixture of low correlation values associated with weak or unreliable edges in the connectome and a sparse set of reliable connections. Consequently, we propose to use alternative thresholding strategy based on the model fit using pseudo-False Discovery Rates derived on the basis of the empirical null estimated as part of the mixture distribution.

We evaluate the method on synthetic benchmark fMRI datasets where the underlying network structure is known, and demonstrate that it gives improved performance with respect to the alternative methods for thresholding connectomes, given the canonical thresholding levels. We also demonstrate that mixture modeling gives highly reproducible results when applied to the functional connectomes of the visual system derived from the n-back Working Memory task in the Human Connectome Project. The sparse connectomes obtained from mixture modeling are further discussed in the light of the previous knowledge of the functional architecture of the visual system in humans. We also demonstrate that with use of our method, we are able to extract similar information on the group level as can be achieved with permutation testing even though these two methods are not equivalent. We demonstrate that with both of these methods, we obtain functional decoupling between the two hemispheres in the higher order areas of the visual cortex during visual stimulation as compared to the resting state, which is in line with previous studies suggesting lateralization in the visual processing. However, as opposed to permutation testing, our approach does not require inference at the cohort level and can be used for creating sparse connectomes at the level of a single subject.

## Highlights

• Sparse functional connectomes are useful in analyzing and interpreting fMRI data.
• We propose thresholding by means of mixture modeling and control of FDR.
• We benchmark the approach on synthetic fMRI data against established methods.
• We apply the method to the resting state and working memory task datasets from HCP500.
• Results are reproducible on synthetic data and interpretable on experimental data.

## Introduction

Functional connectivity (FC) characterizes temporal correlations between signals in the nodes or regions-of-interest (ROIs) in the neuronal network. In functional Magnetic Resonance Imaging (Smith et al., 2013) (fMRI), this concept is used in many contexts. FC serves to study the (co)activity in the neuronal networks, and to investigate links between activity in neuronal networks and cognitive abilities (Smith et al., 2015, Finn et al., 2015, Tavor et al., 2016, Smith, 2016, Chauvin et al., 2017) or clinical-behavioural covariates (Lynall et al., 2010, Garrity et al., 2007, Greicius et al., 2007, Soriano-Mas et al., 2009, Rausch et al., 2016, Oldehinkel et al., 2016, Mulders et al., 2015). It is also used to gain insights into hierarchical structures in the brain in rest and cognition (Smith et al., 2015, Bola and Borchardt, 2016), e.g., the hierarchical structure of sensory systems (Arcaro et al., 2015, Merhar et al., 2016).

In fMRI research, functional connectivity is typically operationalized by means of partial correlation (Marrelec et al., 2006). Since any two processes - even in the absence of underlying direct connection - will almost surely retrieve a non-zero partial correlation by chance, the partial correlation matrices should be constrained in order to remove unreliable connections^2^. As indicated in recent studies by van den Heuvel et al. (van den Heuvel et al., 2017), Zalesky et al. (2016). and Ginestet et al. (2011), the choice of thresholding method can influence the amount of weak connections present in the connectomes, which, in turn, yields an effect on the structure and global properties of the sparsified networks. For this reason, the choice of thresholding method can highly influence the results and interpretation of the results in a functional connectivity study.

There are a few leading approaches to the problem of sparsifying functional connectomes in the field.

Firstly, a popular approach to sparsifying functional connectomes is *proportional* thresholding (Achard and Bullmore, 2007, Bassett and Bullmore, 2009, van den Heuvel et al., 2008). In this thresholding scheme, a top percentage of all partial correlation values in a subject-specific functional connectome is selected. The main aim of this approach is to keep the number of connections fixed for all the individuals in order to eliminate the impact of network density on the comparison of graph metrics across groups. This method for sparsifying functional connectomes is currently the most popular approach in the field, which might be due to its simplicity.

Secondly, partial correlation matrix can be estimated with use of regularizers (Bishop, 2006). Regularization techniques impose sparsity on the network by using a loss function that penalizes for the number of non-zero entries in the connectivity matrix so that the weak connections are shrunk to zero. The shrinkage approach is used to drive weak connections to zero and then to accept everything that has not been set to zero as truly existing. Originally, the goal of the regularizing techniques was not thresholding connectomes, however this became one of the practical applications.

Lastly, thresholding can be performed on the basis of connection-specific significance levels obtained from permutation testing (Welch, 1990). Each of the functional connection can then be thresholded at edge-specific threshold level, according to the edge-specific null. Technically, computing significance levels through permutation testing can be done both at the population- or at the single subject level. On the single subject level, thresholds are estimated from the null-distribution of connections generated by breaking correlations between time series. On the other hand, in order to create the null distribution at the population level, region-specific time series are permuted between subjects, and partial correlation is computed for a number of surrogate networks obtained from permutations across subjects. However, in datasets in which signals are autocorrelated - such as fMRI data where the slow haemodynamic induces autocorrelations in the signal - building the null by shuffling the labels between subjects is preferred over permuting samples on the single subject level, as it keeps the autocorrelations intact.^3^ For this reason, the population level approach was also used in the seminal paper by Smith et al. (2011). As a result, in practice, building significance intervals in permutation testing is only possible on the population (and not on the single subject) level in fMRI connectivity research.

In this work, we propose an alternative to the aforementioned methods: thresholding subject-specific connectomes by means of mixture modeling. To our knowledge, only one work has previously applied this method to model sparse resting-state functional connections (Tyszka et al., 2014). Here we provide a thorough investigation of this technique. Mixture modeling differs from permutation testing as the (pseudo)-null is built subject-wise across all possible connections in the connectome, as opposed to estimating connection-wise null distributions via permutation testing. The underlying assumptions here are that (i) the evidence for a non-zero connection are unrelated to the spatial location of the nodes, (ii) that non-zero connections are sparse and that (iii) there is a sufficient number of nodes so that the set of values for non-existing edges in the network can be used to estimate the within-subject null distribution of non-existing connections.

In our approach, mixture modeling is used in order to separate strong connections in the connectome from a pseudo-null, which is a mixture of noise with weak functional connections. We talk about the pseudo-null as in fact, the functional connectomes in the brain most likely have a scale-free distribution rather than being sparse (Eguiluz et al., 2005, van den Heuvel et al., 2008). Therefore, we can only talk about a ’pseudo-null’ which consists of the ’true null’ distribution of functionally disconnected pairs of nodes, and in addition to that, a part of the scale-free distribution which involve connections too weak to be discerned from the noise with any statistical inference methods. As mainly the strong connections are of interest in connectivity studies, this model choice is a justifiable simplification.

Mixture modeling is a valuable alternative as, on the contrary to permutation testing, it allows for creating connectomes both at the single subject- and at the group level. Furthermore, mixture modeling solves the problem relating to subject-specific proportional thresholding as a technique that allows for weak connections to pass the thresholding in some subjects (for instance, in subjects whose individual connectomes have low number of strong connections compared to other subjects) - which, in turn, changes the global properties in the networks. In mixture modeling, this is not the case, as the total number of connections in the sparsified network is not fixed per subject: *strong* and *weak* connections are determined on the basis of the subject-specific distribution of connections, and mixture modeling provides with a natural separation into the two classes.

The mixture modeling approach used here is popular in other contexts in fMRI research, especially as the basis for thresholding Independent Component Analysis-derived maps (Beckmann and Smith, 2004, Beckmann et al., 2005). It is also used in other applications such as GWAS studies in polygenic disorders (Thompson et al., 2015), where a mixture model is fitted to the distributions of effect sizes for all SNPs.

In our study, we validate mixture-model-based thresholding on the benchmark synthetic fMRI datasets (Smith et al., 2011) derived from Dynamic Causal Modeling generative model (Friston et al., 2003, Smith et al., 2011). Furthermore, we apply our thresholding approach to experimental fMRI datasets from the Human Connectome Project (HCP (Essen et al., 2013),) by creating a sparse connectome of the human visual system at rest and under visual stimulation (Barch et al., 2013). We chose the human visual system because this network incorporates one of best known functional architectures in the human brain, and this allows for qualitative comparison between the methods. We used the n-back working memory (WM) task data from the Human Connectome Project (Barch et al., 2013), because this task involves ongoing visual stimulation by presenting objects in the visual field of the participants. Since during the task, objects of a few categories were presented to the subjects, our hypotheses concentrated on connectivity of the areas responsible for object recognition such as the two areas of the lateral occipital cortex: LO1 and LO2 (Silson et al., 2013, Amedi et al., 2001, James et al., 2002). Multiple studies have revealed that these areas respond to objects defined by luminance, texture or motion but not when subjects view only backgrounds of different textures or coherently moving dots (Grill-Spector et al., 1998). Furthermore, object recognition in the visual system is known as a lateralized process (Warrington and Taylor, 1978): the right hemisphere is responsible for perceptual-, and the left hemisphere is responsible for the semantic categorization. Therefore, we test for asymmetry in the responses to the visual stimuli between the left and the right hemisphere, as compared to the resting state connectivity.

In section 2.1, we introduce the operationalization of functional connectivity. In section 2.2, we introduce the mixture modeling procedure. In section 2.3.1, we list the steps undertaken to validate the method on the synthetic benchmark datasets, and in section 3.1 present quantitative results. In section 2.3.2, we describe datasets and the preprocessing pipeline for computing thresholded functional connectomes, both in rest and under visual stimulation, and in section 3.2, we present the results. Finally, in section 4, we discuss the results in the light of the literature on the functional architecture of the visual system, and propose possible applications.

## Materials and methods

### Operationalization of functional connectivity

Functional connectivity between two nodes X, Y in the network is usually operationalized as either Pearson's or partial correlation (Marrelec et al., 2006) between the time series representing activity in the nodes X(t), Y(t). In the fMRI functional connectivity research, partial correlation analysis is preferred as it reflects direct rather than both direct and indirect functional connections between the nodes (Smith et al., 2011). For each pair of nodes in the network, limiting influence of indirect connections is achieved by regressing out the activity from all the other nodes before computing Pearson correlations. For small networks, the regression step can trivially be performed by means of Ordinary Least Squares regression (OLS (Vittinghoff et al., 2005),). For large networks, however, this may be problematic if the number of possible pairs in the network approaches the length of the BOLD time series, given that partialing out secondary time series involves loss in the temporal degrees of freedom. Therefore, in this work, we choose estimating partial correlation between two nodes in the network by means of the inverse covariance (a.k.a. precision matrix (Christensen, 2011)) normalized by the temporal autocorrelation in the two nodes (Christensen, 2011).

### Mixture modeling

In this work, we model the distribution of pair-wise partial correlation scores (transformed into pseudo-Z statistics using the Fisher *r*-to-*Z* transform) as a mixture distribution of weak, or unreliable, connections and a distribution of values obtained from reliable connections. We exclude the main diagonal entries from the partial correlation matrices as they represent self-connections and further do not take inhibition-induced anti-correlations into account.

We model the pseudo-null either using a Gaussian or Laplace distribution. In principle, as partial correlation relates to the second order statistic, both the null and the mixture representing truly existing connections can be characterized by the Fisher-Snedecor *F* distribution (Box, 1953) (in case of the null, it is an *F*-distribution mirrored around zero). Ideally, it should be modeled as such. However, as this distribution does not belong to the exponential family, its parameters are hard to fit with the Expectation Maximization algorithm (EM (Bishop, 2006, Do and Batzoglou, 2008),).

For the distribution of true connections we chose either Gamma or Inverse Gamma distribution. Again, this is a pragmatic choice as they both represent a distribution of positive values from the exponential family. Unlike simple Gamma distributions the Inverse Gamma distribution cannot deflate towards a flat distribution in the estimation process. In the rest of the manuscript, we use the following abbreviations: GG for the Gauss-Gamma mixture, GIG for Gauss-Inverse Gamma, LG for Laplace-Gamma mixture and LIG for Laplace-Inverse Gamma mixture. The details of the probability density and parameters fitted for each of the aforementioned distributions used in the study, are provided in the Supplementary Material 1.

For all competing models, we infer the relevant mixture parameters using the Expectation Maximization algorithm (Bishop, 2006, Do and Batzoglou, 2008, Llera et al., 2016) with the cut-off threshold set to 0.001. The initialization of the EM algorithm is performed the following way. Firstly, the data is standardised before mixture model fitting. Consequently, the null distribution is initialized with mean μ=0.0 and standard deviation STD=1.0. The activation distribution is initialized with mean μ=3.0 and STD=1.0. Such an initialization ensures that the initial activation distribution models a neglectable percentage of samples in the presence of no connection (a Gaussian white noise) and an increasing percentage of samples with increased connectivity. Furthermore, the mixing proportions are initialized using a flat prior. Such initialization provides a near optimal solution that allows us to avoid re-initialization.

After the model fitting, we propose to threshold the connectome matrices on the basis of the pseudo-False Discovery Rate (pFDR) (Efron, 2007). The False Positive Rate (FPR) is the number of falsely rejected null hypotheses among all elements belonging to the null distribution. In contrast to the FPR, FDR takes into account the overall estimated power of the signal in addition to the estimated amount of type 1 errors. By thresholding connectomes based on FDR, the focus shifts towards controlling the relative amount of positive identifications which makes this estimate ideal for conservative estimations concerning the overall architecture of a network since small influences in the network are more prone to be neglected in favor of strong connections which can therefor be understood as part of the network.

In this work, FDR is used to determine the threshold which is then used to sparsify connectomes. FDR is computed as the number of false positives with respect to the number of positively identified partial correlation values. FPR is estimated based on a model fit which represents the null distribution:

(1) $$FDR\left(x\right)=\frac{\int_{x}^{\infty }p_{0}f_{0}\left(t\right)dt}{\frac{1}{\left|\rho \right|}|\rho \left(t\right)\;for\;t\geq x|}$$

where $f_{0}$ denotes probability density function of the null distribution, $p_{0}$ denotes the size of the null distribution in relation to the whole mixture density, |ρ| denotes the overall number of partial correlation values and |ρ(t)fort≥x| denotes the number of partial correlation values bigger than *x*.

This FDR value can be referred to as a *pseudo*-False Discovery Rate, since it depends on the quality of the model fit. This is because FDR can return values higher than 1.0 in areas where the model overestimates the complete mixture distribution. This also means that the FDR function is not necessarily bijective in such cases, resulting in multiple thresholds with the same FDR value. Because of this effect, we choose the *highest* value passing the FDR cutoff as threshold for sparsifying the functional connectivity matrix.

The thresholding is then based on the probability of assignment to the signal component from mixture modeling, as pseudo-FDR refers to the fitted null distribution. Mind that in this approach, the goodness of the fit to the null is what influences the estimate of the threshold at a given value of pFDR. Therefore, the main purpose of fitting the mixture with a signal component in this approach, is to improve the estimate of the null.

### Validation

#### Synthetic benchmark datasets

We first evaluate our approach on synthetic connectome data generated as in (Smith et al., 2011) using the standard Dynamic Causal Modeling (Friston et al., 2003) generative model for the BOLD fMRI responses (Supplementary Material 2). These simulations allow for emulating network dynamics while controlling a variety of experimental conditions, such as the number of nodes in the network, the session duration, the time resolution of the data, the amount of thermal noise added to the BOLD response or the variability in the haemodynamic lags. We chose to validate our approach by reimplementing simulation no 4 from (Smith et al., 2011) which represents the largest network in the benchmark datasets (N=50 nodes). We created a large population of 500 synthetic ’subjects', and performed 10-min long simulation of the BOLD dynamics, with TR=3.0[s], with addition of 1% thermal noise and with the variability in haemodynamic lags of STD=0.5[s].

Following the generation of the simulated BOLD datasets, we calculate the partial correlation network matrices for the synthetic subjects. On these matrices, we evaluate different mixture models, using either Gaussian or Laplacian distributions for the null and either Gamma or Inverse Gamma distributions for the non-null part of the distribution of partial correlation scores. The covariance matrix can be computed with or without regularization, although in case of regularization, the F-distribution is lost. This set of choices (Gaussian vs Laplacian for the pseudo-null/Gamma vs Inverse Gamma for the strong connections/regularization versus no regularization for the covariance matrix), gives 8 possible mixture models. We compared the 8 possible choices by means of Bayesian Information Criterion (BIC (Schwarz, 1978),) in order to determine which mixture best fits the synthetic datasets. Since all the compared versions of mixture modeling use both the same number of data points and free parameters, the ranking between the version achieved with use of BIC would be identical using Akaike's Information Criterion and other methods for evaluating a model fit (AIC (Akaike, 1998),).

Furthermore, we compare the results between mixture modeling and other methods for sparsifying functional connectomes:

1. Empirical precision (EP) with hard thresholding: the sample covariance matrix, inverted, normalized with autocorrelation in order to obtain partial correlation, and then sparsified. Since there is no optimization technique available for the sparsification step, we apply a *hard* threshold. What we mean by this, is setting a fixed value of partial correlation (of 0.0 in this case), at which the connectomes obtained through empirical precision are thresholded.
2. Ledoit-Wolf regularization (LW (Ledoit and Wolf, 2003),) with hard thresholding: the sample covariance matrix is shrunk towards a fixed target matrix before inverting. Among the available options for the target matrices, we chose the *constant correlation model*, in which the shrinkage target is constructed from the average of all the sample correlations together with the vector of sample variances. This choice for the target matrix is advisable for uniform family of variables,^4^ which is the case here, as every time series in the data represents activity in a single ROI. In the implementation of LW regularization used in this work, there are no free parameters with respect to shrinkage (shrinkage parameter is automatically optimized due to a closed form approximation given in (Ledoit and Wolf, 2003)). Then, the sparse covariance matrix needs to be inverted and normalized with autocorrelation, after this operation, it is no longer sparse. Therefore, similarly as in point 1, we apply a hard threshold of 0.0 to these matrices in order to achieve the sparsity. In this way, sparsification is performed twice: before and after inverting the covariance matrix.
3. Permutation testing: creating a sparse estimate of the partial correlation matrix by constructing a null distribution of connections estimated from time series with shuffled subject labels (confidence levels computed on the population level). The partial correlation matrix is then thresholded at a chosen significance level in the light of this null distribution, for each connection independently (so that FPR is controlled independently for each connection in the network).
4. Proportional thresholding: thresholding partial correlation subject-wise, by selecting a top percentage of all the values in the partial correlation matrix. This is a commonly used approach to thresholding functional connectomes in fMRI (Achard and Bullmore, 2007, Rubinov and Sporns, 2010, Bassett and Bullmore, 2009, van den Heuvel et al., 2008), recently broadly discussed in (van den Heuvel et al., 2017). In our study, we chose two levels for proportional thresholding: 5% and 10%. In the synthetic networks used in our study, the true underlying density of connections equals 6.25%. We decided not to fine-tune the chosen proportions to this particular value, because in real-world applications, this fine-tuning is also not possible and an arbitrary threshold is necessary

The aforementioned methods belong to two separate groups. Mixture modeling and permutation testing are procedures fully based on estimating the null from the data, and therefore on the significance estimation. On the other hand, sparsifying connectomes through hard thresholding (obtained with or without regularization, as described in points 1 and 2), or through proportional thresholding as described in point 4, do not allow for controlling significance. However, we decided to compare all these methods in order to give a comprehensive comparison between different modeling approaches present in fMRI literature.

In the analysis, we only include the upper-diagonal values in the connectomes in order not to duplicate the partial correlation values (which would lead to under-estimation of within-component variance). The goodness of the aforementioned methods is assessed by computing and comparing their *mean performances*. The mean performance is given by the percentage of correctly inferred entries of the binarized precision matrix as compared to the ground-truth binary graph adjacency matrix, averaged over all simulated subjects. The respective mean performances of the considered methods are reported for the following parametrizations: hard-thresholding at the value of 0.0, as well as proportional thresholding through selecting the top 5% and 10% of all the connections for EP and LW, and at p=0.05 for permutation testing and pseudo-FDR. In order to make the results from mixture modeling comparable with permutation testing, the significance levels for both these methods were derived on the group level.

Furthermore, we evaluate the methods on subnetworks of the original 50-node network connectivity pattern. This is because while some of the methods - such as inverse covariance with a hard thresholding at 0.0 - are not sensitive to network size, other methods can be sensitive, and the performance can drop off along with decreasing number of nodes in the network. Namely, in mixture modeling, network size can influence the estimation of significance as it affects the quality of the pseudo-null and signal component estimates, whereas in proportional thresholding, setting a low value of proportion (such as top 5%) can result in empty connectivity matrices if the network is small. Therefore, we create smaller networks of sizes between N=5 and N=49 nodes iteratively, by removing one node at a time. In each subnetwork, we keep at least one pair of nodes which were originally connected in the initial N=50, so that there is always at least one true connection in the network. In the process of shrinking the size of the network, the density of connection stays roughly on the same level.

All the codes for mixture modeling and validation in synthetic datasets, are available at GitHub: https://github.com/FabianWalocha/Thresholding-functional-connectomes-by-means-of-Mixture-Modeling-

#### The human visual system

For validation on fMRI datasets, we use the data from 207 unrelated subjects from the HCP500 cohort (Essen et al., 2013). The unrelated subjects were chosen to prevent bias in the estimates of the network matrix due to familiarity status of the participants.

The following two datasets are used in the analysis:

1. Resting state with eyes open. In each subject, the resting state HCP data involves 4×15[min] runs: two scanning sessions on the two consecutive days where the two sessions per day were recoded using different right-to-left (RL), and left-to-right (LR) phase-encoding directions
2. N-back Working Memory (WM) task (Drobyshevsky et al., 2006, Barch et al., 2013). In this task, subjects' working memory and cognitive control were tested in an n-back task paradigm where different types of visual stimuli (faces, places, tools and body parts) were presented in separate blocks. Each run contains 8 task blocks (of 10 trials, 2.5[s] each) and 4 fixation blocks (15[s] each). Half of the blocks involved a 2-back task (respond when the stimulus is the same as the one two trials before), and the other half involved the 0-back task (a cue stimulus is presented at the start of each block, and the subject must respond to presentation of this stimulus during the block). A 2.5[s]-cue indicates the block type (either 2-back or 0-back, plus the target cue in the latter case) at the start of the block. On each trial, the stimulus is presented for 2[s] and followed by 0.5[s] inter-stimulus interval (ITI). The n-back task was performed once per subject and lasted for a total of 5[min], which amounts to a total of 810 3D volumes (405 vol per phase encoding direction)

The n-back task was chosen for comparison against resting state, because this task requires sustained attention and provides an almost permanent stream of the visual stimuli (presented in blocks with ITIs of 0.5[s], which are substantially shorter than the repetition time (TR) of 0.72[s]). Since ITIs are very short in this particular experiment, we used the whole time series from the n-back task.

All subjects were healthy individuals scanned on a 3-T Siemens connectome-Skyra scanner with $100mTm^{-1}$ gradient strength, TR=0.72[s], TE=33.1[ms], flip angle$=52^{\circ }$, BW=2290Hz/Px, in-plane FOV=208x180[mm], 72 slices and spatial resolution of 2[mm], isotropic, in a multiband setup with an acceleration factor of 8, using a 32-channel head coil. Please see (Ugurbil et al., 2013) for the further acquisition details. The preprocessing was performed using the HCP workbench (Marcus et al., 2013), FSL (Jenkinson et al., 2012) and Freesurfer (Fischl et al., 1999). The structural artifacts were removed using Independent Component Analysis and ICA-based X-noisefier (Salimi-Khorshidi et al., 2014, Griffanti et al., 2014). Then, spatial smoothing was applied using an unconstrained 3D Gaussian kernel of FWHM=3[mm].

The data was then parcellated into ROIs with use of the volume-based probabilistic atlas of visual topography by Wang et al. (2015). We chose for using an atlas rather than for defining ROIs by a seed-based analysis (or by any other functional methods) because the visual system is fine grained and the estimation of ROIs obtained this way could be noisy. The atlas was created by employing retinotopic mapping experiments which resulted IN 50 ROIs (25 per hemisphere) in total: 8 ventral–temporal (V1v, V2v, V3v, hV4, VO1, VO2, PHC1, and PCH2), 9 dorsal–lateral (V1d, V2d, V3d, V3A, V3B, LO1, LO2, TO1, and TO2), 7 parietal (IPS0, IPS1, IPS2, IPS3, IPS4, IPS5, and SPL1), and one frontal (hFEF) region. We mapped the probabilistic assignments into ROIs on one 3D map of maximum probability assignments for all the voxels. Since the original atlas has twice higher spatial resolution than the HCP data, this 3D map was subsampled to 91 × 109 x 91 voxels. As known from previous computational studies, mixing signals within ROIs is very detrimental to the connectivity research in fMRI (Smith et al., 2011, Bielczyk et al., 2017a). Therefore in case voxels within a given block of 2 × 2 x 2 voxels belonged to two or more separate ROIs from Wang's atlas, we did not assign any label to that block in the downsampled atlas.

In order to prevent mixing signals between ROIs, we classified each set of 2 × 2 x 2 voxels into a new ROI, but only when all the 8 voxels belonged to the same ROI in the original Wang's atlas. After this scaling, certain voxels at the boundaries between ROIs did not receive new labels and therefore, a few small regions disappeared from further analysis. The following regions were thus discarded: MST (lH,rH), hMT (lH,rH), IPS3(lH,rH), IPS4(lH,rH), IPS5(lH,rH), SPL1 (lH,rH), FEF (lH,rH). The remaining 36 ROIs were included in the analysis.

Then we prepared the data for the analysis in two different ways, for two different purposes:

1. For comparison between sparse connectomes in the resting state and under visual stimulation: we removed the initial 5 frames from the data for each version of the encoding in order to prevent scanning-related artifacts. Then, for the n-back WM task datasets, the BOLD time series from each version of the encoding was normalized, and the two time series were merged into a vector of 800 samples per subject. For the resting state data, the procedure was repeated, except that the BOLD time series was additionally shortened so that it matched the length of the task data (800 samples per subject in total), so that the differences in the distribution of partial correlation values between task and rest are not caused by differences in precision of estimating partial correlation (which depends on the length of the time series).
2. For the test-retest comparison, the data was *rearranged*: the LR encoding from the first day was concatenated with RL encoding from the second day, and vice versa.

Subsequently, functional connectivity was estimated, and mixture modeling was performed in the same fashion as in synthetic datasets (Section 2.2): by fitting 8 possible versions of the mixture model, and comparing against each other by means of BIC.

Finally, for the test-retest comparison we used Index of Overlap in order to compare the results from day 1 against day 2, both in the resting state and in the n-back WM task. Furthermore, to establish the level of inter-subject variability between sparse connecomtes obtained from each method, we computed the intraclass correlation coefficient (ICC (Shrout and Fleiss, 1979, Herting et al., 2017, Vetter et al., 2017),) as a standard tool in the functional connectivity research (Fiecas et al., 2013), for the resting state and for the task datasets from day 1.

## Results

### Synthetic benchmark datasets

**Fig. 1:**
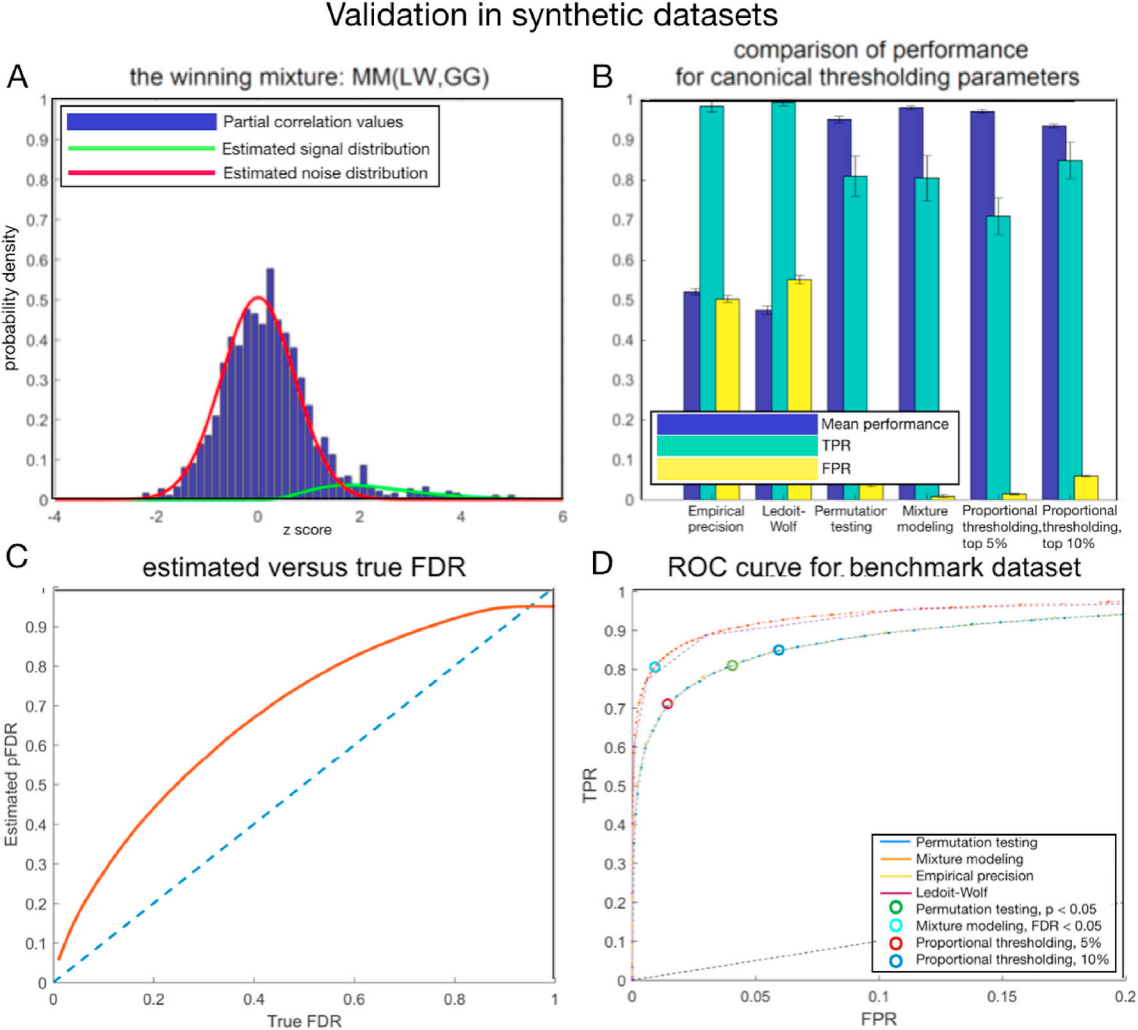
Validation on synthetic benchmark datasets. **A**: The best mixture modeling fit: Gauss-Gamma mixture computed on partial correlation estimated with Ledoit-Wolf regularization. Red: the pseudo-null. Green: the component representing strong connections. **B**: The mean performance across 500 synthetic subjects, for the canonical parameters (thresholding at 0 for empirical precision and LW, p=0.05 for permutation testing, FDR=0.05 for mixture modeling). In order to make the results from mixture modeling comparable with permutation testing, the significance levels for both these methods were derived on the group level. Mean performance: mean ratio of correctly identified links (connection/lack of connection). TPR - true positive rate. FPR - false positive rate. Mixture modeling gives the best trade-off between TPR and FPR, therefore the overall performance is the highest. **C**: The estimated FDR in a function of the true FDR. The FDR estimation with use of mixture modeling is conservative. **D**: The ROC curve for the synthetic dataset, comparing mixture modeling MM(LW,GG) with all other methods. The FPR range was clipped to [0,0.2] because the values outside this range are, typically, not of interest in neuroimaging studies. Circles denote the canonical values of the thresholding parameter (p=0.05 for permutation testing, FDR=0.05 for mixture modeling, 5% and 10% cut-off for proportional thresholding). Mixture modeling gives the highest AUC, and hard-thresholding partial correlation obtained from Ledoit-Wolf regularized covariance matrix is the runner-up.

Among 8 possible versions of the mixture modeling, Gauss-Gamma mixture based on the inverse covariance computed with LW regularization, which we further refer to as MM(LW,GG), achieved the lowest BIC score (BIC = 3282.2). The fit of this winning misture to the synthetic datasets is presented in Fig. 1 A. The runner-up model according to the BIC score is a Gauss-Inverse Gamma mixture based on the inverse covariance computed with LW regularization (hereon called MM(LW,IG), BIC = 3308.6). We present the detailed results of this comparison in the Supplementary Material 3.

In Fig. 1 B, we present the comparison between mixture modeling and other methods for thresholding connectomes, given the canonical thresholds (0 for empirical precision and for LW, p=0.05 for permutation testing, pFDR=0.05 for mixture modeling, 5% and 10% cut-off for proportional thresholding). Mixture modeling gives the best trade-off between True- and False Positive Rate, therefore the overall performance is the highest.

In Fig. 1 C, we present the estimated FDR in a function of the true FDR. As we can observe, the FDR estimation with use of mixture modeling is conservative. In Fig. 1 D, we compare the ROC curve between mixture modeling and other methods. In mixture modeling, we vary the value of thresholding value of pseudo-FDR. In permutation testing, we vary the thresholding p-value. In empirical precision and Ledoit-Wolf regularized empirical precision, we vary the value of the hard threshold. In proportional thresholding, we vary the proportion of filtered connections per subject. Among all the compared methods, AUC for the mixture modeling is the highest. The results for the canonical values of the thresholding parameter (p=0.05 for permutation testing, FDR=0.05 for mixture modeling, 5% and 10% cut-off for proportional thresholding) are presented in circles.

**Fig. 2:**
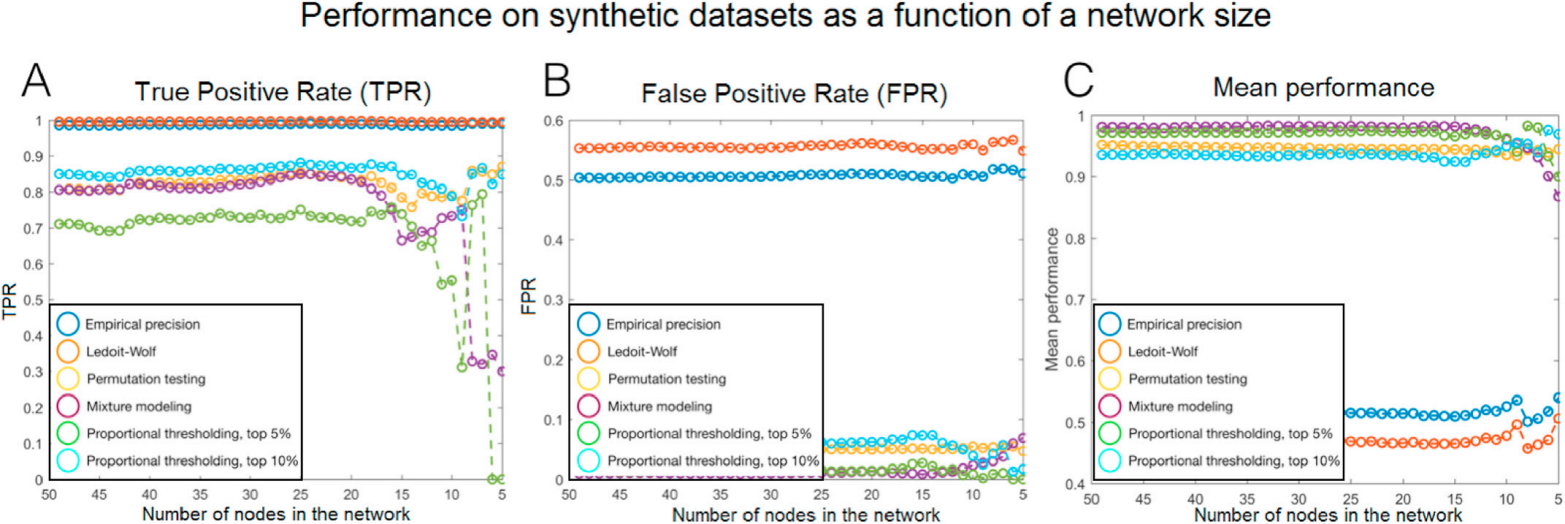
The evaluation on synthetic datasets, for network sizes between 5 and 50 nodes. The performance of Mixture Modeling is stable across networks bigger than N=15 nodes, and decreases for network sizes between N=10 and N=15 nodes. Its overall performance for large networks also minimally higher than all the other methods, including proportional thresholding at 5%, which is close to the true network density (6.25%).

In Fig. 2, we present the analysis of the influence of the network size on the performance, for network sizes varying between 5 and 50 nodes. The results follow the expectations: for mixture modeling and proportional thresholding at low percentage of 5%, the results fall off along with decreasing network sizes. The performance of Mixture Modeling is stable across networks bigger than N=15 nodes, and slowly decreases for network sizes between N=10 and N=15 nodes. Its overall performance is also minimally higher than all the other methods, including proportional thresholding partial correlation at 5%, which is close to the true network density, fluctuating around 6.25%. Ledoit Wolf and Empirical precision thresholded at zero give significantly worse results than the rest of the methods, for similar reasons as when evaluated on the original network (Fig. 1B): they are not conservative enough and therefore, give a very high False Positive Rate.

### The human visual system

In both task and rest, the mixture model with lowest BIC was Gauss-Gamma mixture based on the inverse covariance computed with LW regularization, MM(LW,GG). (BIC = 1515.3, BIC = 1663.1 for rest and task, respectively). For the resting state data, the runner up was Gauss-Inverse Gamma mixture based on the inverse covariance computed with LW regularization, MM(LW,IG) for the resting state data with (BIC = 1536.2). For the task data, the runner up was Gauss-Gamma mixture based on unregularized inverse covariance (BIC = 1668.9). The detailed results of the comparison are presented in Supplementary Material 3.

**Fig. 3:**
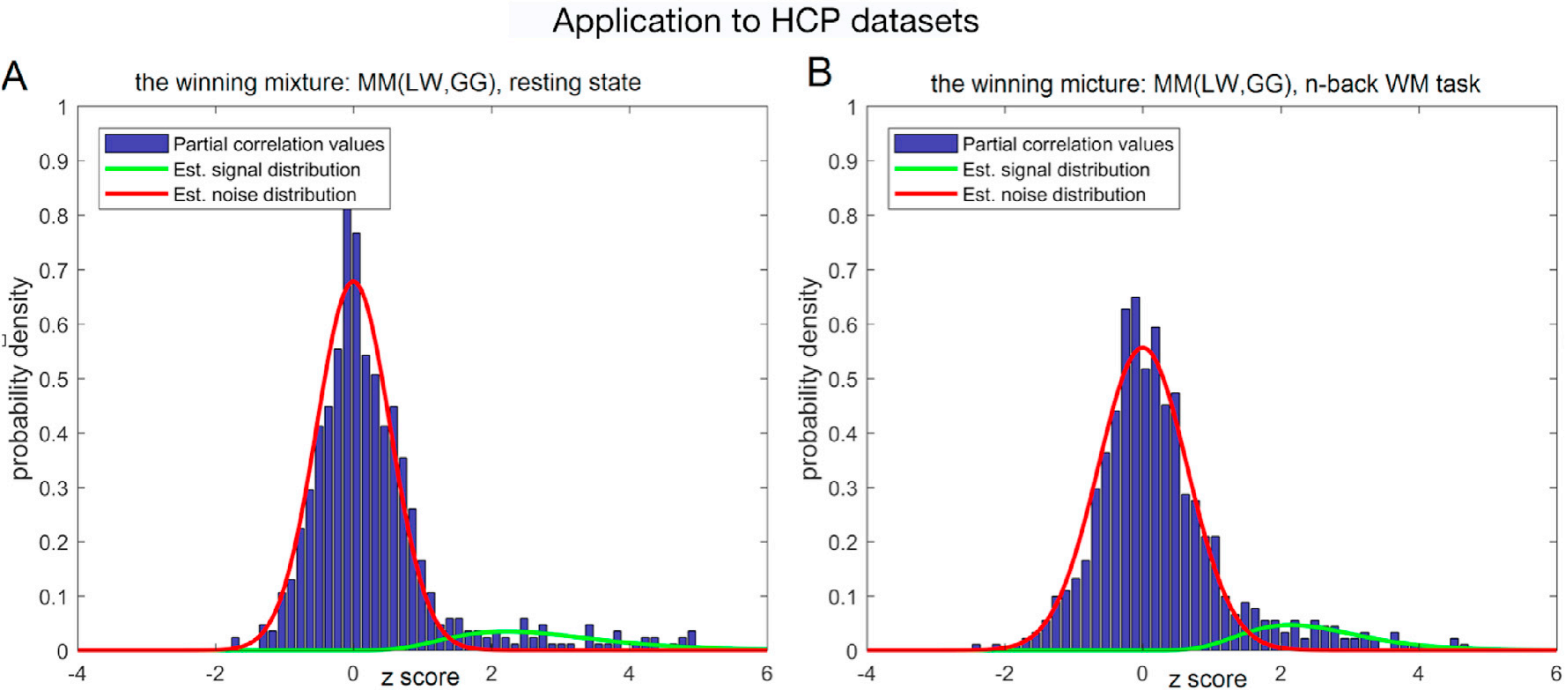
The model fit with the lowest BIC: Gauss-Gamma fit based on the inverse of the Ledoit-Wolf regularized covariance. Red: the pseudo-null. Green: the component representing connections. For convenience, the tails of the distribution were truncated outside the range [−4.0,+6.0] on the plot.

Fig. 3 presents the best mixture fit to the normalized values of the partial correlation values from 207 unrelated subjects from the HCP datasets (Essen et al., 2013), in the resting state and under the visual stimulation.

The mean numbers of connections found per subject, in both resting state and in the n-back WM task, are presented in Table 1. As mixture modeling and permutation testing thresholded at the canonical thresholds are more conservative than other methods, the group connectome for these two methods have less overall number of connections per subject than in case of the other two methods. Moreover, interestingly, all the methods report a *decrease* in the mean number of connections per subject in the task versus rest.

**Table 1:** The mean density of connections per subject, in the resting state and in the n-back WM task, at the canonical thresholds. The mixture modeling gives significantly sparser connectomes at the canonical value of the threshold than competitive methods.

no	method	Rest	Task	Difference
1	Empirical precision	0.574 (±0.017)	0.558 (±0.013)	−0.016
2	Ledoit-Wolf	0.584 (±0.018)	0.568 (±0.017)	−0.016
3	Permutation testing	0.115 (±0.013)	0.092 (±0.013)	−0.022
4	MM(LW,GG)	0.089 (±0.009)	0.068 (±0.012)	−0.021
5	Proportional thresholding, top 5%	0.048 (±0.0)	0.048 (±0)	−0.0
6	Proportional thresholding, top 10%	0.098 (±0.0)	0.098 (±0.0)	−0.0

In Table 2, the Index of Overlap in the resting state and in the n-back WM task (day 1 against day 2) are presented. Mixture modeling is the most conservative method, therefore the associated Index of Overlap scores are highest (and close to 100). In Table 3, the ICC scores for similarity between subject-specific sparse connectomes (computed for scanning on day 1 only) in the resting state and in the n-back WM task are presented. Again, the most conservative methods give the highest ICC scores. Moreover, ICC scores over 0.50 are at the upper end of results reported in the literature (Shehzad et al., 2009).

**Table 2:** Index of Overlap between the two scanning sessions: mean per subject + standard deviation in the brackets. Naturally, methods giving the sparsest connectomes also result in the highest Index of Overlap. The group difference in Index of Overlap between task and rest is insignificant for all the methods.

no	method	Rest	Task	Difference
1	Empirical precision	60.15 (±2.25)	66.09 (±2.33)	+5.94
2	Ledoit-Wolf	61.53 (±2.24)	67.75 (±2.45)	+6.22
3	Permutation testing	93.79 (±1.08)	94.15 (±1.66)	+0.36
5	MM(LW,GG)	96.03 (±0.89)	97.53 (±4.87)	+0.38
6	Proportional thresholding, top 5%	97.66 (±0.54)	97.53 (±0.65)	−0.13
7	Proportional thresholding, top 10%	93.47 (±0.84)	93.21 (±0.97)	−0.26

**Table 3:** ICC scores in the resting state and in the n-back WM task across subjects (day 1 only). The most conservative methods give the highest ICC scores, exceeding 0.5. The difference between rest and task is not pronounced for any of the methods.

no	method	Rest	Task	Difference
1	Empirical precision	0.0945	0.0976	+0.0031
2	Ledoit-Wolf	0.1012	0.1055	+0.0043
3	Permutation testing	0.5181	0.4506	−0.0675
5	MM(LW,GG)	0.6192	0.5795	−0.0397
6	Proportional thresholding, top 5%	0.6218	0.5831	−0.0387
7	Proportional thresholding, top 10%	0.5377	0.4828	−0.0549

**Fig. 4:**
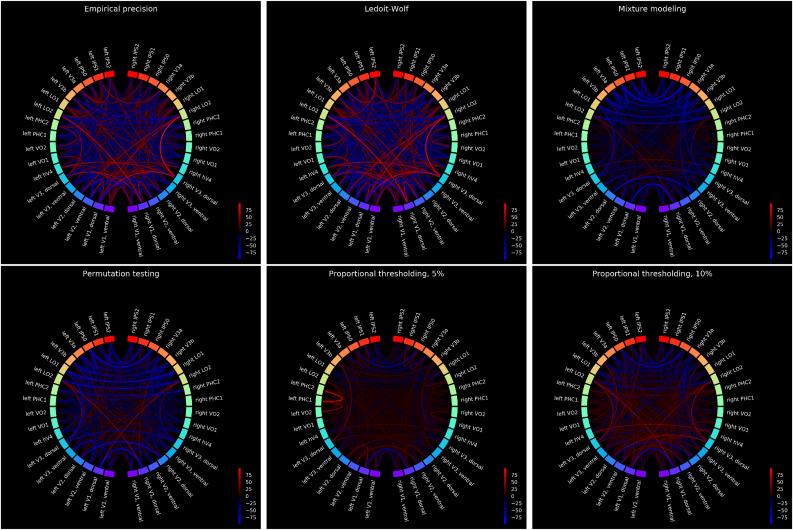
The group difference between resting state and n-back WM task, for six methods including mixture modeling, permutation testing and proportional thresholding. In order to make the results from mixture modeling comparable with permutation testing, the pFDR was derived on the group level. The group statistic is provided on the basis of the set of subject-specific, individual sparse connectomes obtained with canonical parameters (hard thresholding at 0 for empirical precision with and without regularization, p=0.05 for permutation testing, FDR=0.05 for mixture modeling, 5% and 10% cut-off for proportional thresholding). Then, for each connection, we calculated the number of subjects in which this connection was found under the visual stimulation, and subtracted the number of subjects in which this connection was found in the resting state. The intensity of connections on the circular plots refers to this *relative* difference: red color means that a given connection appeared as significant in more subjects during the WM task as compared to resting state, whereas intense blue relates to connections which appear in less subjects under visual stimulation rather than during rest. The homologous regions are placed against each other (on the left, versus on the right side of the plots). The figures present the raw results.

**Fig. 5:**
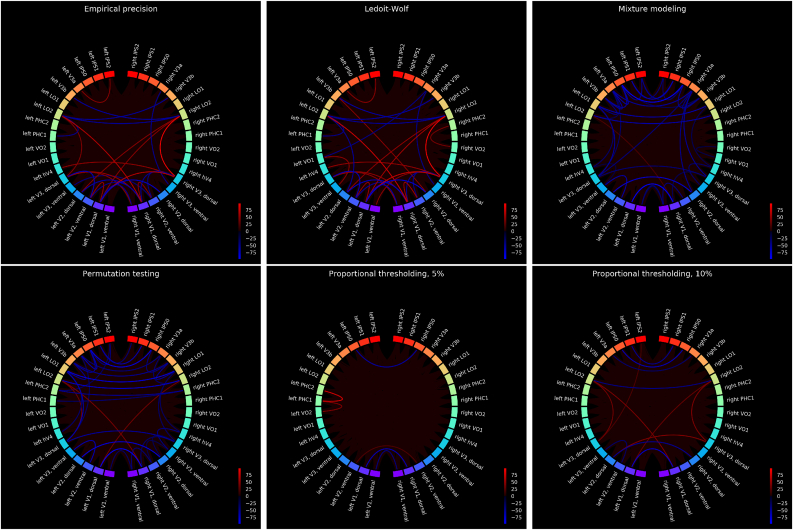
Results as in Fig. 4, corrected for multiple comparison with non-parametric testing (Mann-Whitney *U* test at the significance level p=0.01 with a Bonferroni correction).

Fig. 4 and Fig. 5 present a *group* comparison between the WM task and the resting state (session 1, time series shortened to 800 samples in each subject, as described in Section 2.3.2), uncorrected and Bonferroni-corrected, respectively. In these plots, we provide with a group statistic on the basis of the set of subject-specific, individual sparse connectomes: for each connection, we calculated the number of subjects in which this connection was found under the visual stimulation, and subtracted the number of subjects in which this connection was found in the resting state. The intensity of connections on the circular plots refers to this *relative* difference: red color means that a given connection appeared as significant in more subjects during the WM task as compared to resting state, whereas intense blue relates to connections which appear in less subjects under visual stimulation rather than during rest. In the circular plots, the homologous regions are placed against each other (on the left, versus on the right side of the plots). This plotting routine highlights the homologous connections (which is of primary importance in our results).

In Fig. 4, Fig. 5, we compare the six methods previously evaluated in the synthetic datasets, for the canonical value of the thresholding parameter.

What we observe from nonparametric testing is that systematic differences between functional connectivity at rest and under visual stimulation can be observed with use of all the methods (at a very conservative significance level p=0.01 with a Bonferroni correction). For both permutation testing and mixture modeling, results are similar: there is a dominant effect of decoupling between homotopic areas, especially high in the visual hierarchy, including LO1 and LO2 areas. In particular, functional decoupling between higher order visual areas in parietal cortex (IPS0, IPS1, IPS2) is revealed, along two dimensions: between the homologous counterparts and between the nearby regions within each ipsilateral part of the parietal cortex. Proportional thresholding at the 5% threshold on the other hand, does not reveal such an effect, but instead, suggests unilateral functional coupling between left PHC1 and PHC2 during visual stimulation.

Mixture modeling and permutation testing give similar results when applied to experimental fMRI datasets. However, as these two methods threshold the connectome along different dimensions (across subjects in permutation testing and across connections in mixture modeling), the p-values for these two methods are not directly comparable. Therefore, next to the comparison for the canonical p-value, we also provide with a full comparison between mixture modeling and permutation testing for the full range of possible thresholds, a visualization available under the address: http://mmthresholding.byethost6.com/.^5^

Additionally, in Supplementary Material 5, we present a comparison between the results obtained for permutation testing with the null obtained in two ways: through shuffling labels between subjects, and through permuting the time series within each subject's BOLD time series. By making this comparison, we demonstrate that the method to create the null in permutation testing has a major influence on the group results in the study. We confront these results with mixture modeling, in order to demonstrate that unlike in permutation testing, results obtained with mixture modeling based on confidence intervals derived on subject- and on population level, are similar.

In Table 4, we present the percentages of nonzero connections in Fig. 4, Fig. 5.

**Table 4:** A percentage of non-zero links in Fig. 4, Fig. 5.

no	method	Fig. 4	Fig. 5
1	Empirical Precision	94.92	3.97
2	Ledoit-Wolf	93.49	4.60
3	Permutation testing	94.60	5.56
5	MM(LW,GG)	71.90	6.03
6	Proportional thresholding, top 5%	38.89	0.95
7	Proportional thresholding, top 10%	86.35	2.06

## Discussion

### Discussion of the results

In this work, we propose a mixture-model-based approach to estimating sparse subject-specific functional connectomes. Mixture modeling allows for setting a threshold at a user-defined level of pseudo-FDR, which is a measure for separation between strong and weak connections within every subject's individual connectome. In the synthetic datasets, where the ground truth is known, our approach gives best trade-off between TPR and FPR (for the canonical parameter of pseudo-FDR=0.05). Furthermore, on experimental fMRI HCP datasets, we observe increased ICC for mixture modeling as compared to other methods (this effect, however, might be due to the fact that mixture modeling gives the most conservative thresholding).

We propose this approach as an *alternative* to the most popular methods, proportional thresholding and permutation testing. Even though proportional thresholding is currently the most popular approach in the field, in this work, we paid special attention to comparing our approach to permutation testing, as they are both based on a concept of estimating FPR, either in a nonparametric or in a parametric way. According to our results, Mixture Modeling yields a better AUC than permutation testing when applied to synthetic datasets, and similar results when applied to HCP datasets. However, mixture modeling and permutation testing are *not* equivalent as they sparsify the functional connectome along a different dimension: connection-wise and across the population in permutation testing, versus across the functional connectome (subject-wise or population-wise) in mixture modeling. Furthermore, given one value of pseudo-FDR for mixture modeling and *p* for permutation testing, the sparse connectome is sparser for mixture modeling, because the FDR is always lower or equal than FPR it is derived from (Eq. (1)). Therefore, these two methods are not directly comparable.

We propose using mixture modeling as an alternative to permutation testing for functional connectivity research in cohorts of a very few subjects, such as translational psychiatry paradigms. It is possible as long as the network is large enough to build a distribution from the entries of the partial correlation matrix. According to our results in synthetic datasets, network sizes of N=15 or more are sufficient for the application of the mixture modeling approach. However, in real world applications, this will also depend on the signal-to-noise ratios in the data and the density of the underlying ’true’ functional connectome.

In this work, we demonstrate one potential application of mixture modeling for thresholding connectomes on the example of the human visual system. Comparing the influence of visual stimulation on the functional connectome, mixture modeling and permutation testing reveal a very similar pattern of (de)-activation under visual stimulation on the group level. In both rest and cognition, the group functional connectome obtained from these two methods reflects the current state of the knowledge upon the functional architecture of the visual system (Supplementary Material 4, Fig. 10). We observe a clear functional hierarchy (Haak and Beckmann, 2017), reflecting the histological (Hagmann et al., 2003) and functional (Wandell et al., 2007, Grill-Spector and Malach, 2004) findings on the architecture of the visual system in humans (and also animal studies in cats and macaques (Felleman and Essen, 1991, Tootell et al., 2003, van Essen and Maunsell, 1983)). In our study, we observe that the primary visual cortex V1 occupies the bottom of the functional hierarchy (Felleman et al., 1997), and sends strong projections to the secondary visual cortex (V2). We also observe projections from V2 to V3, however projections from V2 to higher visual areas reported in the literature (Grill-Spector and Malach, 2004), are not visible.

We also observe interhemispheric symmetry in the group functional connectome, with almost identical pattern of functional connectivity in both hemispheres (Fig. 10). The functional coupling between the homologous areas is, however, more pronounced in the resting state than under visual stimulation (Fig. 5), and the difference is most pronounced in the high levels of the visual hierarchy. This functional decoupling in high visual areas under visual stimulation can relate to the lateralization theory of visual perception (de Schotten et al., 2011), which states that visual processing in two hemispheres is different (Courtney et al., 1996, Güntürkün et al., 2000).^6^

Lastly, we can observe a strong coupling between subsequent levels of the visual hierarchy within the dorsal and within the ventral visual stream. The ventral stream, known as the ”what pathway” or ”perception pathway” (Lerner et al., 2001), is thought to be responsible for object recognition, and leads from the striate cortex towards the temporal lobe (Goodale and Milner, 1992, Mishkin et al., 1983). The dorsal stream a.k.a. the ”where pathway” or ”action pathway”, encodes for spatial location of objects (Goodale and Milner, 1992, Mishkin et al., 1983), and leads from the striate cortex towards the parietal lobe. In our study, we can clearly delineate both streams within V1, V2 and V3. Furthermore, the functional decoupling between hemispheres under the visual stimulation is much more pronounced in the dorsal stream than in the ventral stream (Fig. 5). In the experimental paradigm implemented in HCP datasets (Barch et al., 2013), the visual stimuli were presented at the fixation point (in the center of the visual field), which means that the same amount of visual stimulation was delivered to both hemispheres. Therefore, the functional decoupling between the contralateral parts of the ”where” pathway under the visual stimulation suggest that the lateralization in the visual processing starts at a very early stage of the visual processing, possibly even in V1. Since the visual system has the functional architecture of a convolutional network (Güclü and van Gerven, 2015, Wandell et al., 2007), this asymmetry amplifies in the high levels of visual hierarchy.

### Limitations of the method

In our approach, we do not take inhibition into account. Inhibition in the brain is primarily found on two levels of organization. Firstly, in the small scale: single inhibitory cells form local inhibitory networks, densely interconnected within single brain regions (Fino and Yuste, 2011). Secondly, in the global scale: large scale resting state networks are often anticorrelated with each other (Fox et al., 2005, Uddin et al., 2009, Chai et al., 2012) (for instance, the Default Mode Network is anticorrelated with various task-positive networks). Our work is dedicated to modeling networks on a *meso-scale* level: within single Resting State Networks (Damoiseaux et al., 2006, Smith et al., 2009). On this level of description, anticorrelations between the nodes of the network are rarely encountered. In particular, in our study on the visual system, we observe no anticorrelations in the data, neither in rest nor under visual stimulation.

For this reason, we believe that in the aforementioned applications, mixture modeling of just the two components - pseudo-null and one component representing connections - is valid. In case of possible extensions of this research to the full brain research, there might be a necessity of adding a third component, representing anticorrelations between the resting state networks. Furthermore, we choose to use a parametrized mixture model in order to fit the null (using either Gaussian or Laplace distribution), but in principle, one could also use the Efron approach (Efron, 2007) of fitting a spline to the total distribution and then fitting an empirical null to that spline fit.

The length of the time series is also an important aspect of the mixture modeling as it affects the width of the null distribution: the shorter the time series, the broader the null and the more uncertainty in the estimation of the signal component. In our case, the estimation of partial correlation matrix was performed on a high quality datasets with 800 samples. The long time series and high data quality may explain why ICC scores are as high in this study, for all the examined methods (Table 2, in the range of 0.5 as opposed to 0.15 encountered in the literature (Fiecas et al., 2013)). In addition, we used a single session of the n-back WM task, and sectioned the data into two time series, which does not capture the day-to-day variability in the functional connectivity during cognition.

The last issue is the intrinsic limitations of the synthetic datasets used in this study. Firstly, in the synthetic datasets, we simulated the DCM generative model highly acknowledged in the community (Smith et al., 2011, Friston et al., 2003). Whether this model gives a satisfactory representation of the experimental fMRI datasets, remains an open question. However, out of 8 possibilities, we obtained the same mixture achieved the lowest BIC score, both with respect to the true HCP datasets, and with respect to the synthetic datasets, which suggests that the distributions of connections for the synthetic and true datasets are fairly comparable. Secondly, in the experimental fMRI datasets, the preprocessing pipeline can influence the connectivity studies to a large extent (Aurich et al., 2015, Marrelec and Fransson, 2011). In this study, we chose the high quality, single-site datasets from the HCP500 cohort (Essen et al., 2013), and we used the standard FIX motion regression (Salimi-Khorshidi et al., 2014, Griffanti et al., 2014). Also, we chose for a high quality parcellation of the visual cortex by Wang et al. (2015), and for the highly potent very potent visual working memory task which known to give strong activation across the brain, especially in the visual cortex (Barch et al., 2013). Therefore, we believe that we chose a pipeline that gave us the highest chances for obtaining reliable results for the group task versus rest comparison on the experimental datasets.

### Further research

The application of mixture modeling to threshold connectomes in the visual system, as presented in this manuscript, is a proof of concept. The visual system was chosen because we are able to formulate and test hypotheses on the basis of the prior knowledge upon the anatomical and functional architecture of the visual system. In the future, this approach can also be applied for thresholding connectomes in other mesoscale networks.

Furthermore, in this work, we constructed and compared group connectomes in the task in during resting state, and we performed this comparison connection by connection rather than by looking into higher order statistics of these connectomes, graph theoretical properties etc. As known from the literature, thresholding procedure can highly influence these global properties in the networks (Zalesky et al., 2016), therefore a follow up study on this aspect of the thresholding should also be performed.

Lastly, as the previous research suggests that the false positives have a profound effect on the network organization, the future research might include other implementations of mixture modeling with use of other component distributions, more robust to false positives etc.

### Conclusions

Building functional connectomes of the large scale networks, in rest and under cognitive stimulation, is a large and still developing subfield of fMRI research. In many contexts, it is beneficial or even mandatory to sparsify the functional connectome. E.g., in the graph theoretical studies, sparse connectomes allow for studying global properties of the functional networks in health (Bullmore and Sporns, 2009) and disease (van den Heuvel et al., 2010, Zhang et al., 2011a, Zhang et al., 2011b). Certain graph theoretical measures such as topological overlap between two nodes in the network or Rentian scaling (wiring efficiency) are based on the sparse connectomes only (Brain Connectivity Toolbox (Bullmore and Sporns, 2009),). Next, there is a class of models characterizing the dynamics in the human connectome by means of Ising models (Stramaglia et al., 2017) which require sparsity. Furthermore, recent developments in the field of causal inference in fMRI involve a two-step inference procedure. In such a procedure, connections are spotted in the network by creating a sparse functional connectome (Patel et al., 2006, Hyvärinen and Smith, 2013, Bielczyk et al., 2017b), and the causal discovery is performed pairwise. Sparse connectomes are also useful in multiple other disciplines that involve graph theory, i.e., in social networks (Watts and Strogatz, 1998, Mislove et al., 2007), protein-protein, protein-DNA and gene-gene interactions (Eisen et al., 1998, Pellegrini et al., 2004).

The selection of available methods for sparsifying functional connectomes is still growing. For instance, among the recent developments in the field, there are new, fused estimators for thresholding functional connectomes (Zille et al., 2017), dedicated to comparing two different groups of functional connectomes, i.e., connectomes derived for a group of children versus adults, or for one group of subjects undergoing two different cognitive tasks. Given the comparison between the methods on synthetic benchmark datasets, our results agree with the previous studies suggesting that the thresholding technique has a major influence on the type of information contained in the sparse connectome, and therefore should be approached with care. For instance, in proportional thresholding, the threshold is often an arbitrary choice (Achard and Bullmore, 2007, Rubinov and Sporns, 2010, Bassett and Bullmore, 2009, van den Heuvel et al., 2008). As discussed in a recent theoretical study by van den Heuvel et al. (van den Heuvel et al., 2017), and also previous studies (Nichols et al., 2017, van Wijk et al., 2010, Alexander-Bloch et al., 2010), including weak and therefore unreliable connections (in subjects with overally weak functional connectivity) to the connectome has an effect on the organization of the functional network and graph metrics.

Since the mixture modeling gives a trade-off between a data-driven approach (as in permutation testing) and subject-specific thresholding (as in proportional thresholding), we propose using mixture modeling for thresholding connectomes as an alternative to existing approaches.

## Disclosure/Conflict-of-interest statement

The authors declare that the research was conducted in the absence of any commercial or financial relationships that could be construed as a potential conflict of interest. JCG has acted as a consultant to Boehringer Ingelheim in the last 4 years, but is not an employee or shareholder of this company.

## Author contributions

NZB, FW, PWE and CFB designed the study. FW and PWE conducted the study in collaboration with NZB and AL. NZB drafted the manuscript. FW wrote the codes for mixture modeling and validation in synthetic and experimental fMRI datasets. NB wrote the codes for DCM generative model after (Smith et al., 2011). CFB, AL, KVH, JKB and JCG critically revised the work.

## Funding

NZB has received funding from the European Community's Seventh Framework Programme (FP7/2007–2013) under grant agreement no 305697 (OPTIMISTIC), the European Community's Seventh Framework Programme (FP7/2007–2013) under grant agreement no 278948 (TACTICS) and European Union's Seventh Framework Programme for research, technological development and demonstration under grant agreement no 603016 (MATRICS). FW has received financial and organizational support from the ERASMUS + programme for an internship which made this collaboration possible. CFB gratefully acknowledges funding from the Wellcome Trust UK Strategic Award [098369/Z/12/Z]. CFB is supported by the Netherlands Organisation for Scientific Research (NWO-Vidi 864-12-003). KVH is supported by the Netherlands Organisation for Scientific Research (NWO 016.Veni.171.068). CFB and KVH gratefully acknowledge funding from the Wellcome Trust UK Strategic Award [098369/Z/12/Z].

## Appendix A Supplementary data

YNIMG_14616.docx p.no 25-29 YNIMG_14616.docx p.no 25-29

## Footnotes

2 Neurobiologically speaking, one may argue that the brain is highly integrative and therefore, the connections represented by low values of a partial correlation should arguably be referred to as *weak* - in the sense of indistinguishable from the noise.

3 We also demonstrate the effect of breaking autocorrelations on the results of permutation testing in Supplementary Material 5.

4 The original version of the Ledoit-Wolf regularization was designed for the stock market analysis, where different data coming from different instruments, such as stocks and bonds, is often fed into the same model (Ledoit and Wolf, 2003).

5 In the dynamic plots, we can sometimes observe single connections disappearing and reappearing again. This is caused by the fact that the number of subjects for whom a given connection was found in rest and under visual stimulation, grows at different speeds along with the thresholding parameter.

6 which is believed to have certain adaptive value.
