# Supplementary material for: Thresholding functional connectomes by means of mixture modeling
Source: Neuroimage. 2018 May 1;171:402–14. doi: 10.1016/j.neuroimage.2018.01.003 (PMC5981009; doi:10.1016/j.neuroimage.2018.01.003)
Supplement: YNIMG_14616.docx p.no 25-29 [file mmc1.docx]

**Supplementary Materials**

**1. Mixture components**

In the study, we used four following distributions, each of them represented by a set of two free parameters as listed below:

1. Gaussian:

$$p(x|\mu,\sigma)=\frac{1}{\sqrt{2\pi\sigma^{2}}}e^{-\frac{(x-\mu)^{2}}{2\sigma^{2}}}$$

where μ - mean of the distribution, σ - standard deviation of the distribution.

2. Laplace:

$$p(x|\mu,\theta)=\frac{1}{2\theta}e^{-\frac{|x-\mu|}{\theta}}$$

where μ - mean of the distribution, θ - a scale parameter.

3. Gamma:

$$p(x|k,\theta)=\frac{\theta^{-k}}{\Gamma(k)}x^{k-1}e^{-\frac{x}{\theta}}$$

where k - a shape parameter, θ - a scale parameter.

4. Inverse Gamma:

$$p(x|k,\theta)=\frac{\theta^{k}}{\Gamma(k)}x^{-k-1}e^{-\frac{\theta}{x}}$$

where k - a shape parameter, θ - a scale parameter.

**2. Dynamic Causal Modeling generative model**

In this work, we use the original, single node per region DCM (Friston et al., 2003, Smith et al., 2011). This model operationalizes the generation of BOLD response from the neuronal networks across two levels: non-observable neuronal level and the observable haemodynamic level.

The latent neuronal dynamics is described by the simple differential relationship:

$$\frac{d\overset{\to}{z}}{dt}=A\overset{\to}{z}\left( t-\tau\right)+C\overset{\to}{u}\left( t \right)+\overset{\to}{\sigma}(t)$$

where z denotes the temporary activity across all nodes, u denotes binary inputs (trains of on- and off- states in our case), A denotes the adjacency matrix of effective connectivity and C denotes connections from (experimental) inputs to the nodes, τ denotes a lag in the neuronal communication, and σ denotes the level of stochasticity on the neuronal level. In our network setup, the modulatory connectivity does not play a role for the research question, therefore we set all the modulatory connections B from the original DCM model (Friston et al., 2003) to zero. The connectivity (a.k.a. adjacency) matrix A contains self-inhibition in every node as originally proposed in (Friston et al., 2003) and, since our simulations refer to simulation no 4 in the benchmark synthetic datasets (Smith et al., 2011), the connectivity matrix A is highly sparse and modular.

Additionally, we use small, biologically plausible time lags of in the communication between areas as also implemented in (Smith et al., 2011), therefore the simulated network becomes a system of delayed differential equations in fact (Bocharov and Rihan, 2000).

In this context, the stochastic term σ, implemented as a Wiener process, represents neuronal innovations which are not the part of the communication between nodes of the investigated network (Daunizeau et al., 2012). It can either represent intrinsic dynamics in the given node (other than inhibition), or input from areas outside the investigated network. Strictly speaking, these innovations are not a ’noise’ (which would mean stochasticity added to the neuronal time series on the top of the simulated dynamics), but rather a background neuronal dynamics which cannot be explained by the given model. However, for the sake of simplicity we will refer to σ as noise in the text below.

The observational level is given by the classic model for the haemodynamic response, referred to as Balloon-Windkessel model (Buxton et al., 1998, Friston et al., 2003), is described node-wide, and for every node i it is described by the dynamics of four biophysiological variables as follows:

$$\frac{ds_{i}}{dt}=z_{i}\left( t \right)-\kappa_{i}s_{i}\left( t \right)-\gamma_{i}(f_{i}\left( t \right)-1)$$

$$\frac{df_{i}}{dt}=s_{i}\left( t \right)$$

$$\lambda_{i}\frac{dv_{i}}{dt}=f_{i}\left( t \right)-v_{i}^{1/\alpha}\left( t \right)$$

$$\lambda_{i}\frac{dq_{i}\left( t \right)}{dt}=f_{i}\left( t \right)\frac{E\left( f_{i}\left( t \right),\rho_{i} \right)}{\rho_{i}}-v_{i}^{\frac{1}{\alpha}-1}\left( t \right)q_{i}(t)$$

where s_i_(t) - vasodilatory signal, f_i_(t) - inflow, v_i_(t) - blood volume, q_i_(t) - deoxyhemoglobin content, E(f,ρ) = 1 - (1-ρ)^1/f^. The model involves five node-specific constants: κ - rate of signal decay, γ - rate of flow-dependent elimination, λ - haemodynamic transit time, α - Grubb’s exponent, ρ - resting oxygen extraction fraction.

Then, the following expression describes the outcome BOLD response:

$$y\left( t \right)=V_{0}\left( 7\rho_{i}\left( 1-q_{i}\left( t \right) \right) \right)+2\left( 1-\frac{q_{i}\left( t \right)}{v_{i}\left( t \right)} \right)+(2\rho_{i}-0.2)(1-v_{i}(t))$$

where denotes the resting blood volume fraction.

Inputs to the network were simulated as in Smith et al., 2011: as independent trains of on- and off-states with time resolution of 5[ms]. The probability of state switches was governed by a Poissonian process of a mean on-state duration of 2.5[s], and a mean off-state duration of 7.5[s]. In our simulations, similarly as in Smith et al., 2011, the variance of the neuronal noise σ(t) was set to 0.05 of the input value, which means that the SNR is very high (in the range of SNR=20).

**3. Comparison between different versions of mixture modeling**

We compared the possible versions of mixture modeling by evaluating its BIC values both on the synthetic benchmark dataset and on the two HCP datasets (the resting state and the n-back WM task). The possibilities are:

1. computing empirical precision (EP) with or without LW regularization

2. Gaussian or Laplace distribution to model the pseudo-null

3. Gamma or Inverse Gamma to model the distribution of connections

which gives a total of 8 possible versions.

In Figure 6, we compare the fits on the synthetic dataset. We obtain the following set of results:

1. Μethods based on empirical precision matrices with LW-regularization perform better than methods based on empirical precision matrices without LW-regularization (mean BIC = 3338.5 / 3451.1, respectively). The difference is significant (p < ε in ranksum test, where ε - machine precision).

2. Μethods fitting Gaussian to pseudo-null perform better than methods fitting a Laplace distribution (mean BIC = 3352.5 / 3436.5, respectively). The difference is significant (p < ε in ranksum test).

3. Methods fitting Gamma distribution to model the distribution of connections perform better than methods fitting Inverse Gamma distribution to model the distribution of connections (mean BIC = 3387.3 / 3396.1, respectively). The difference is significant (p = 2.4 * 10^-6^ in ranksum test).

As a result, MM(LW,GG) mixture is the mixture with the lowest BIC score in the synthetic dataset.

Figure 6: Comparison of BIC for all the 8 mixture modeling methods on the synthetic dataset. **A:** all 8 versions. **B:** methods based on LW-regularized precision matrices perform better than mixture modeling methods based on empirical precision matrices (BIC=3338.5, BIC=3451.1 respectively). **C:** methods fitting a Gaussian to the pseudo-null perform better than methods fitting a Laplace distribution (BIC=3352.5, BIC=3436.5 respectively). **D:** methods fitting Gamma distribution to model the distribution of true connections perform better than mixture modeling methods fitting Inverse Gamma distribution (BIC=3387.3, BIC=3396.1 respectively).

In Figure 7, we compare the fits via BIC on the resting state HCP datasets. We obtain the following set of results:

1. Methods based on empirical precision matrices with LW-regularization perform better than methods based on empirical precision matrices without LW-regularization (mean BIC = 1538.0/1545.6, respectively). The difference is not significant (p = 0.0649 in ranksum test)

2. Methods fitting Gaussian to pseudo-null perform better than methods fitting a Laplace distribution (mean BIC = 1529.9 / 1553.7, respectively). The difference is significant (p = 1.5 * 10^-8^ in ranksum test)

3. Methods fitting Gamma distribution to model the distribution of connections perform better than methods fitting Inverse Gamma distribution to model the distribution of connections (mean BIC = 1533.8 / 1549.8, respectively). The difference is significant (p = 1.2 * 10^-4^ in ranksum test)

As a result, MM(LW,GG) mixture is also the mixture with the lowest BIC score in the resting state HCP dataset.

Figure 7: Comparison of BIC for all the 8 mixture modeling methods on the resting state HCP dataset. **A:** all 8 versions. **B:** methods based on LW-regularized precision matrices perform better than mixture modeling methods based on empirical precision matrices (BIC=1529.9, BIC=1553.7 respectively). **C:** methods fitting a Gaussian to the pseudo-null perform better than methods fitting a Laplace distribution (BIC=1529.9, BIC=1553.7

respectively). **D:** methods fitting Gamma distribution to model the distribution of true connections perform better than mixture modeling methods fitting Inverse Gamma distribution (BIC=1533.8, BIC=1549.8 respectively).

In Figure 8, we compare the fits via BIC on the HCP datasets from the n-back WM task. We obtain the following set of results:

1. Methods based on empirical precision matrices with LW-regularization perform better than methods based on empirical precision matrices without LW-regularization (mean BIC = 1669.9 / 1676.1, respectively). The difference is not significant (p = 0.985 in ranksum test)

2. Methods fitting Gaussian to pseudo-null perform better than methods fitting a Laplace distribution (mean BIC = 1660.0 / 1686.2, respectively). The difference is significant (p = 8.1*10^-11^ in ranksum test)

3. Methods fitting Gamma distribution to model the distribution of connections perform better than methods fitting Inverse Gamma distribution to model the distribution of connections (mean BIC = 1667.7 / 1678.3, respectively). The difference is significant (p = 0.0059 in ranksum test)

As a result, MM(LW,GG) mixture is also the mixture with the lowest BIC score in the n-back WM HCP dataset.

Figure 8: Comparison of BIC for all the 8 mixture modeling methods on visual WM task HCP dataset. **A:** all 8 versions. **B**: methods based on LW-regularized precision matrices perform better than mixture modeling methods based on empirical precision matrices (BIC=1669.9, BIC=1676.1 respectively). **C:** methods fitting a Gaussian to the pseudo-null perform better than methods fitting a Laplace distribution (BIC=1660.0, BIC=1686.2 respectively). **D:** methods fitting Gamma distribution to model the distribution of true connections perform better than mixture modeling methods fitting Inverse Gamma distribution (BIC=1667.7, BIC=1678.3 respectively).

**4. Group sparse connectomes in the resting state and under cognitive stimulation**

Figure 9: A ’group sparse connectome’ for the conventional methods with canonical thresholds: a summary of the number of subjects for whom the given connection survived the thresholding. **A:** resting state. **B:** n-back WM task. In all cases, the strong signatures of inter-hemispheric communication as well as signatures of the ventral- and dorsal streams are preserved.

Figure 10: A ’group sparse connectome’ for permutation testing and for the mixture modeling methods: a summary of the number of subjects for whom the given connection survived the thresholding. **A:** resting state. **B:** n-back WM task. Both permutation testing and mixture modeling are more conservative and give sparser group connectome than empirical precision and Ledoit-Wolf thresholded at 0 (Figure 9).

Figure 11: A ’group sparse connectome’ for proportional thresholding methods: a summary of the number of subjects for whom the given connection survived the thresholding. **A:** resting state. **B:** n-back WM task. The group connectomes are sparse, as in permutation testing and mixture modeling (Figure 10).

**5. Comparison of permutation testing results obtained from the null built on the subject- versus on population level**

Figure 12: Comparison between results obtained from subject- and cohort level, for mixture modeling and permutation testing. In this example, the analysis is the same as in the results presented in Fig. 5: thresholding at p = 0.05 or pFDR = 0.05, counting connections over the population, subtracting the number of subjects for whom a given connection was found during task versus rest, and Mann-Whitney U test at p = 0.01 with a Bonferroni correction. In mixture modeling, thresholding at the pFDR derived for each subject separately (for the subject-specific distribution of partial correlation values), and at the cohort level (for the joint distribution of partial correlation values in the whole population), gives similar results. On the contrary, in permutation testing, thresholding at the p level derived for each subject separately (by permuting samples in the time series), and at the cohort level (by shuffling time series between subjects), gives different results.
